# Supplementary material for: Dorsal premammillary projection to periaqueductal gray controls escape vigor from innate and conditioned threats
Source: eLife. 2021 Sep 1;10:e69178. doi: 10.7554/eLife.69178 (PMC8457830; doi:10.7554/eLife.69178)
Supplement: Transparent reporting form [file elife-69178-transrepform1.docx]

***eLife’s* transparent reporting form**

We encourage authors to provide detailed information *within their submission* to facilitate the interpretation and replication of experiments. Authors can upload supporting documentation to indicate the use of appropriate reporting guidelines for health-related research (see [EQUATOR Network](http://www.equator-network.org/%20)), life science research (see the [BioSharing Information Resource](https://biosharing.org/)), or the [ARRIVE guidelines](http://www.plosbiology.org/article/info:doi/10.1371/journal.pbio.1000412) for reporting work involving animal research. Where applicable, authors should refer to any relevant reporting standards documents in this form.

If you have any questions, please consult our Journal Policies and/or contact us: [editorial@elifesciences.org](mailto:editorial@elifesciences.org).

**Sample-size estimation**

- You should state whether an appropriate sample size was computed when the study was being designed
- You should state the statistical method of sample size computation and any required assumptions
- If no explicit power analysis was used, you should describe how you decided what sample (replicate) size (number) to use

Please outline where this information can be found within the submission (e.g., sections or figure legends), or explain why this information doesn’t apply to your submission:

Sample sizes for human and mouse experiments were determined based on comparisons to similar published papers.

This information can be found in the “Behavioral Cohort Information” section in the Methods.

**Replicates**

- You should report how often each experiment was performed
- You should include a definition of biological versus technical replication
- The data obtained should be provided and sufficient information should be provided to indicate the number of independent biological and/or technical replicates
- If you encountered any outliers, you should describe how these were handled
- Criteria for exclusion/inclusion of data should be clearly stated
- High-throughput sequence data should be uploaded before submission, with a private link for reviewers provided (these are available from both GEO and ArrayExpress)

Please outline where this information can be found within the submission (e.g., sections or figure legends), or explain why this information doesn’t apply to your submission:

The information below can be found in the “Behavioral cohort information” section in the Methods.

Initial behavioral characterization of the assays (Figure 1) was replicated 3 times, with cohorts containing 10, 10 and 12 mice (32 in total).

PMd cell body fiber photometry experiments (Figure 2) were replicated twice with cohorts containing 7 and 8 mice (15 in total).

Miniscope experiments (Figures 3 and 4) were replicated twice, with cohorts of 4 and 5 mice (9 in total)

Chemogenetics experiments (Figure 5) were replicated twice (cohort 1 with 10 controls and 6 hM4Di mice and cohort 2 with 9 controls and 5 hM4Di mice).

ChR2 experiments (Figure 6) were done once, with 5 YFP and 4ChR2 mice.

dlPAG fiber photometry experiments (Figure 7) were replicated twice, with cohorts of 4 and 5 mice (9 in total)

amv body fiber photometry experiments (Figure 7) were replicated once with 6 mice.

PMd-dlPAG optogenetic projection inhibition experiments (Figure 8) were replicated twice. Both cohorts had 12 controls and 6 arch mice.

PMd-amv optogenetic projection inhibition experiments (Figure 8) were replicated twice. Both cohorts had 6 controls and 9 arch mice.

Appropriate fluorophore-only expressing mice were used as controls for chemogenetic and optogenetic experiments.

For fMRI data, a cohort of 48 human subjects was used only once.

Each mouse was only exposed to each assay once, as defensive behavior assays cannot be repeated. Thus, there are no technical replicates. No outliers were found or excluded. All mice and humans were used.

**Statistical reporting**

- Statistical analysis methods should be described and justified
- Raw data should be presented in figures whenever informative to do so (typically when N per group is less than 10)
- For each experiment, you should identify the statistical tests used, exact values of N, definitions of center, methods of multiple test correction, and dispersion and precision measures (e.g., mean, median, SD, SEM, confidence intervals; and, for the major substantive results, a measure of effect size (e.g., Pearson's r, Cohen's d)
- Report exact p-values wherever possible alongside the summary statistics and 95% confidence intervals. These should be reported for all key questions and not only when the p-value is less than 0.05.

Please outline where this information can be found within the submission (e.g., sections or figure legends), or explain why this information doesn’t apply to your submission:

All the requested statistical information is in the figure legends.

(For large datasets, or papers with a very large number of statistical tests, you may upload a single table file with tests, Ns, etc., with reference to sections in the manuscript.)

**Group allocation**

- Indicate how samples were allocated into experimental groups (in the case of clinical studies, please specify allocation to treatment method); if randomization was used, please also state if restricted randomization was applied
- Indicate if masking was used during group allocation, data collection and/or data analysis

Please outline where this information can be found within the submission (e.g., sections or figure legends), or explain why this information doesn’t apply to your submission:

For chemogenetic and optogenetic experiments, mice in each cage were randomly allocated to control (mcherry or YFP -expressing mice) or experimental conditions (hM4Di, ChR2 or Arch –expressing mice). Data collection was done blinded to treatment group in mice.

For human fMRI data and mouse neural activity recordings, all data were obtained from subjects in identical conditions, and thus they were all allocated to the same experimental group. There were no experimentally controlled differences across mice and there were no “treatment groups”.

This information can be found in the “Behavioral cohort information” section in the Methods.

**Additional data files (“source data”)**

- We encourage you to upload relevant additional data files, such as numerical data that are represented as a graph in a figure, or as a summary table
- Where provided, these should be in the most useful format, and they can be uploaded as “Source data” files linked to a main figure or table
- Include model definition files including the full list of parameters used
- Include code used for data analysis (e.g., R, MatLab)
- Avoid stating that data files are “available upon request”

Please indicate the figures or tables for which source data files have been provided:

All data was uploaded to dryad and all code was uploaded to github (see Materials and Methods, ‘Data and Code Availability’ section).

Custom analysis scripts are available at

<https://github.com/schuettepeter/PMd_escape_vigor>.

Data is available at

<https://datadryad.org/stash/share/dYuSl2nnXsyi0nTDjCDeHR08gwW7paFL4Eo3TmF_aH4>
